# Cationic Imidazolium-Urethane-Based Poly(Ionic Liquids) Membranes for Enhanced CO_2_/CH_4_ Separation: Synthesis, Characterization, and Performance Evaluation

**DOI:** 10.3390/membranes14070151

**Published:** 2024-07-09

**Authors:** Guilherme Dias, Laura Rocca, Henrique Z. Ferrari, Franciele L. Bernard, Fernando G. Brandão, Leonardo Pereira, Sandra Einloft

**Affiliations:** 1 School of Technology, Pontifical Catholic University of Rio Grande do Sul (PUCRS), Porto Alegre 90619-900, RS, Brazil; guilherme.dias@acad.pucrs.br (G.D.); l.rocca@edu.pucrs.br (L.R.); henrique.zucchetti@edu.pucrs.br (H.Z.F.); franciele.bernard@pucrs.br (F.L.B.); 2 Post-Graduation Program in Materials Engineering and Technology, Pontifical Catholic University of Rio Grande do Sul (PUCRS), Porto Alegre 90619-900, RS, Brazil; 3 Petrobras/CENPES, Ilha do Fundão Qd. 07, Rio de Janeiro 21941-915, RJ, Brazil; fernando.brandao@petrobras.com.br (F.G.B.); lspereira@petrobras.com.br (L.P.)

**Keywords:** CO_2_ capture, poly(ionic liquids), ionic liquids, polyurethane, permeability

## Abstract

The escalating emissions of CO_2_ into the atmosphere require the urgent development of technologies aimed at mitigating environmental impacts. Among these, aqueous amine solutions and polymeric membranes, such as cellulose acetate and polyimide are commercial technologies requiring improvement or substitution to enhance the economic and energetic efficiency of CO_2_ separation processes. Ionic liquids and poly(ionic liquids) (PILs) are candidates to replace conventional CO_2_ separation technologies. PILs are a class of materials capable of combining the favorable gas affinity exhibited by ionic liquids (ILs) with the processability inherent in polymeric materials. In this context, the synthesis of the IL GLYMIM[Cl] was performed, followed by ion exchange processes to achieve GLYMIM variants with diverse counter anions (NTf_2_^−^, PF_6_^−^, and BF_4_). Subsequently, PIL membranes were fabricated from these tailored ILs and subjected to characterization, employing techniques such as SEC, FTIR, DSC, TGA, DMA, FEG-SEM, and CO_2_ sorption analysis using the pressure decay method. Furthermore, permeability and ideal selectivity assessments of CO_2_/CH_4_ mixture were performed to derive the diffusion and solubility coefficients for both CO_2_ and CH_4_. PIL membranes exhibited adequate thermal and mechanical properties. The PIL-BF_4_ demonstrated CO_2_ sorption capacities of 33.5 mg CO_2_/g at 1 bar and 104.8 mg CO_2_/g at 10 bar. Furthermore, the PIL-BF_4_ membrane exhibited permeability and ideal (CO_2_/CH_4_) selectivity values of 41 barrer and 44, respectively, surpassing those of a commercial cellulose acetate membrane as reported in the existing literature. This study underscores the potential of PIL-based membranes as promising candidates for enhanced CO_2_ capture technologies.

## 1. Introduction

In the context of contemporary technological progress, a noticeable increase in atmospheric CO_2_ emissions originating from the utilization of fossil resources has emerged [1,2]. In response, the imperative to develop new technologies or refine existing ones to improve environmental impacts has become increasingly evident. A particularly promising avenue of investigation lies in CO_2_ capture. This technological approach garners attention due to its applicability to major emission sources and its potential for the recovery and repurposing of CO_2_ for other industrial processes [2,3]. Currently, diverse techniques for CO_2_ capture exists, among which amine solutions and polymeric membranes stand out as exemplary materials employed for this purpose [4]. The amine-based capture method, which involves the chemical absorption of CO_2_, is widely used in emission plants for handling combustion gases and in natural gas sweetening [5,6,7,8,9]. Despite its cost effectiveness, this approach presents challenges such as the generation of undesirable by-products, the formation of corrosive acids leading to the degradation of the internals of the reactor, volatility, and the high energy costs associated with CO_2_ recovery, thus diminishing its attractiveness over time [10]. On the other hand, the utilization of membranes in CO_2_ separation demonstrates significant potential due to their adaptability to high CO_2_ concentrations and their more compact structural requirements compared to amine solutions [11]. Ionic liquids (ILs) constitute a class of organic salts composed of a cation and an anion, being an organic cation and an organic or inorganic anion [4,12,13,14]. Characterized by a melting temperature below 100 °C, ILs possess several properties expected of effective CO_2_ capture materials, including low vapor pressure, non-flammability, thermal stability, low corrosiveness, minimal decomposition rates, lower recovery costs, and environmental compatibility vis-à-vis organic solvents [4,12,15,16]. ILs have attracted attention in the domain of combustion gas capture due to their enhanced CO_2_ affinity relative to gas mixtures containing N_2_ and CH_4_. However, ILs present specific attributes that impede their immediate adoption in CO_2_ capture plants, such as high production costs and complexity compared to amine solutions, as well as high viscosity resulting in suboptimal CO_2_ sorption/desorption rates [4]. Polymerized ionic liquids or poly(ionic liquids) (PILs) represent a distinct class of materials with broad applicability across various scientific domains [17,18,19,20,21]. Typically composed of a polymeric structure hosting an ionic liquid unit for each polymer repeat unit, PILs offer superior CO_2_ sorption/desorption kinetics, thus conferring a significant advantage over ILs [12,22,23,24,25]. Condensation polymerization stands out as a common method for PIL synthesis. Researchers, including Bernard 2019, da Luz 2021, and Morozova 2020 [26,27,28] have successfully synthesized PILs and assessed their properties. Materials such as PTMG (polytetramethylene glycol), PCD (polycarbonate diol), and PCL (polycaprolactone) serve as illustrative examples of polyols used as starting reagents along with diisocyanates for PIL synthesis [26,27,29]. In the case of polyurethane (PU)-based cationic PILs, the respective ILs in the form of diols are employed, thus incorporating the IL cation into the polymer chain as documented in the literature [26,29,30]. This study endeavors to synthesize and evaluate the CO_2_/CH_4_ separation potential of PU-based cationic PIL membranes. The IL GLYMIM[Cl] was synthesized, followed by ion exchange processes to obtain GLYMIM with different counter anions (NTf_2_^−^, PF_6_^−^ and BF_4_^−^). These ILs were used as diols in the synthesis of PILs, aiming to tailor polymeric chains with high affinity for CO_2_, thereby producing membranes with improved selectivity.

## 2. Materials and Methods

### 2.1. Materials

For the synthesis of hydroxyl-functionalized ionic liquids (ILs), N-methylimidazole (Sigma-Aldrich, Burlington, MA, USA), 3-chloro-1,2-propanediol (Sigma-Aldrich), ethyl acetate (Neon Química, São Paulo, Brazil), lithium bis(trifluoromethanesulfonyl)imide (LiNT_2_F, Sigma-Aldrich, USA), lithium tetrafluoroborate (LiBF_4_, Sigma-Aldrich, USA), and sodium hexafluorophosphate (NaPF_6_, Sigma-Aldrich, USA) were used. To obtain the PILs, polycarbonate diol (PCD, Mn = 2000 g/mol, Bayer, Berlin, Germany), hexamethylene diisocyanate (HDI, 99%, Merck, Darmstadt, Germany), dibutyltin dilaurate (DBTDL, Miracema Nuodex, Campinas, Brazil), methyl ethylketone (MEK, 99%, Mallinckrodt, Hazelwood, MO, USA) and the obtained ILs glyceryl-N-methylimidazolium chloride [GLYMIM][Cl], and derivatives ([GLYMIM][Cl], [GLYMIM][NT_2_F], ([GLYMIM][BF_4_] and ([GLYMIM][PF_6_]) were used.

### 2.2. Synthesis of Hydroxyl-Functionalized Ionic Liquids

The synthesis of glyceryl-N-methylimidazolium chloride was conducted as described in the literature [25,31,32]. The reaction was carried out continuously in a glycerin bath with constant magnetic stirring at a temperature of 70 °C. During the first hour of the reaction, 10 mL (0.11 mol) of 3-chloro-1,2-propanediol was slowly added dropwise to a solution containing 12 mL (0.15 mol) of N-methylimidazole. The reaction conditions were maintained for 72 h. 

Subsequently, the reaction mixture was removed from the heat and washed six times with ethyl acetate. The resulting material was then placed in a glycerin bath to maintain a constant temperature of 70 °C and dried under vacuum for 24 h.

**Figure 1:**
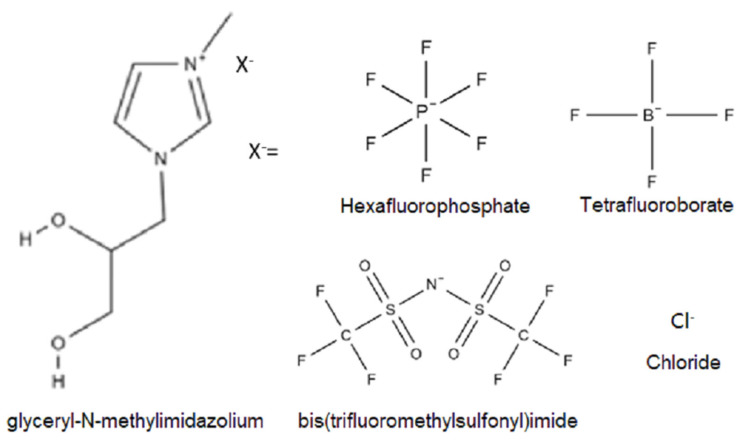
ILs structure.

The product was stored under a nitrogen atmosphere, resulting in a yellow oil [25,31,32]. Ion exchanges were performed in acetonitrile by reacting [GLYMIM]Cl with the respective salts: LiNT_2_F to obtain [GLYMIM]NT_2_F, LiBF_4_ to form [GLYMIM]BF_4_, and NaPF_6_ to form [GLYMIM]PF_6_. It should be noted that the reagents used in the ion exchange process ([GLYMIM]Cl, LiBF_4_, LiNT_2_F, and NaPF_6_) are soluble in acetonitrile, while the by-products, LiCl and NaCl, are not soluble. This allowed for easy separation of the by-products through simple filtration [25,32,33,34]. The obtained structures can be observed in Figure 1.

AgNO_3_ tests were carried out until no significant amount of white precipitate was observed, confirming satisfactory removal of the by-product. Vacuum drying was then performed to remove any excess solvent and moisture.

### 2.3. Cationic Poly(Ionic Liquids)

**Figure 2:**
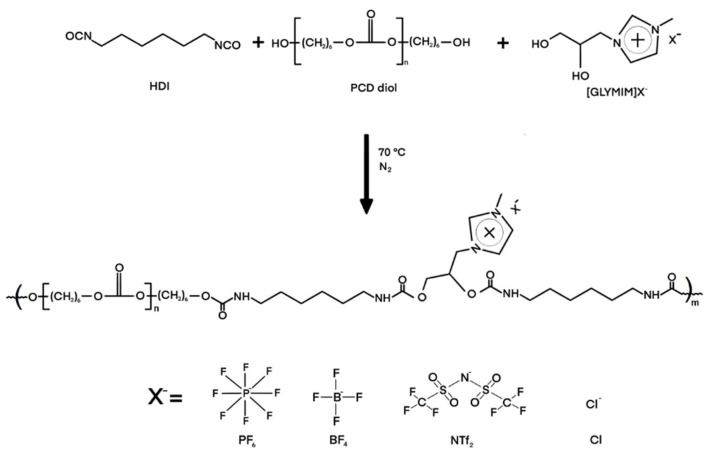
Structures of the cationic PILs.

Initially, under constant stirring, the polyol PCD (0.04 mol) was melted in a five-neck reactor, the DBTDL catalyst (0.1% by weight) was added, and 50 mL of methyl ethyl ketone was added. After reaching the reaction temperature (70 °C), the diisocyanate (0.047 mol) was added to form the prepolymer. Then, dialcohol ([GLYMIM]Cl) (0.11 mol) and diisocyanate (0.11 mol) were added to form the PIL. The completion of the reaction is indicated by the disappearance of the free NCO band (around 2270 cm^−1^) in the infrared spectrum. The synthesized PILs were labeled as PLIX, where X represents the counteranion (Cl, NT_2_F, PF_6_, or BF_4_). Figure 2 shows the structural formula of a cationic PIL.

### 2.4. Syntheses of PU

The PU preparation process was the same as the method used for the synthesis of cationic poly(ionic liquid); however, [GLYMIM]X, was not added, using an NCO/OH molar ratio of 1.1.

### 2.5. Preparation of Dense Membranes 

Membranes were prepared using the casting or solvent evaporation method with thickness between 150 and 250 μm. Initially, a 20% *w*/*w* solution of PU or PIL was prepared by dissolving 2 g of PIL in 10 mL of methyl ethylketone by magnetic stirring and heating (50 °C) until the PIL was completely dissolved. The solution was then applied to a flat surface, such as a glass plate or a Petri dish, followed by solvent evaporation at room temperature for five days.

### 2.6. Characterization 

Hydroxyl-functionalized ILs and synthesized PILs were characterized by Fourier transform infrared spectroscopy (FTIR, PerkinElmer Spectrum 100 spectrometer, PerkinElmer, Waltham, MA, USA) in transmission mode in the range of 4000–650 cm^−1^ to verify their structures. The synthesized ILs were also characterized by nuclear magnetic resonance (NMR) using a Bruker Ascend 400 model NMR spectrometer, Bruker, Billerica, MA, USA.

The molar masses of the PIL membranes and their distribution were determined by gel permeation chromatography (GPC), with chromatograms obtained using the isocratic HPLC pump-1515 chromatograph (LabMakelaar Benelux, Zevenhuizen, The Netherlands) coupled with a Waters Instruments 2412 (Waters Corp, Milford, CT, USA) refractive index detector and THF as eluent.

Scanning electron microscopy with field emission (FEI Inspect F50, FEI Company, Hillsboro, OR, USA) analyses were performed in secondary electrons (SE) mode to evaluate the surface of the composite membranes.

Differential scanning calorimetry (DSC, TA Instruments Q20, TA Instruments, New Castle, DE, USA) was used to determine the glass transition temperature (Tg), melting temperature (Tm), and crystallization temperature (Tc) of the PILs. Tests were performed with two heating ramps and one cooling ramp in the range of −90 to 200 °C at 10 °C/min under an inert nitrogen atmosphere.

The thermal stability of the PILs was assessed through thermogravimetric analysis (TGA) using a TA Instruments Q600 model instrument, ranging from room temperature to 600 °C with a heating rate of 10 °C/min under a nitrogen atmosphere.

Mechanical analyses were performed in triplicate according to ASTM D822 standard [35] on a TA Instruments Q800 (TA Instruments, New Castle, DE, USA) apparatus to determine Young’s moduli and obtain stress–strain curves. 

### 2.7. CO_2_ Sorption Capacity

The pressure decay technique was used to determine the CO_2_ sorption capacity. A detailed description of the sorption apparatus and measurement procedure can be found in our previous works [26,27]. Samples (1.0 g) were placed in the sorption chamber and degassed under vacuum (10^−3^ mbar) for 1 h at room temperature before the test began. CO_2_ sorption experiments were carried out at 30 °C at different equilibrium pressures (1 bar and 10 bar).

### 2.8. CO_2_ Permeability and Ideal CO_2_/CH_4_ Selectivity

The permeation and selectivity for CO_2_ and CH_4_ of the PLI membranes were evaluated in a system consisting of two plates joined in a 4 cm diameter hole (Figure S1), where the membrane is inserted. Vacuum was applied to the membrane and the system before gas feed. CO_2_ or CH_4_ was fed at a pressure of 4 bar. At the bottom, a pressure transducer computed the amount of gas passing through the membrane over time (dp/dt). The permeate gas was assumed to exhibit ideal behavior; therefore, normal temperature and pressure conditions were used. Permeability was determined from the slope (dp/dt) of the linear portion of pressure versus time using Equation (1):

(1) $$\frac{P}{l}=\frac{dp}{dt}\left(\frac{V_{System}}{A\;∆p}\right)\left(\frac{T_{CNTP}}{T\;P_{CNTP}}\right)\;\;\;$$

where l is the membrane thickness, P is permeability, Δp is the pressure difference across the membrane, A corresponds to the membrane area, V_System_ is the volume of the permeation cell, T is the ambient temperature, and T_CNTP_ and P_CNTP_ are, respectively, the temperature and pressure at normal conditions [36]. The ideal selectivity was calculated from Equation (2) by dividing the CO_2_ permeability by the CH_4_ permeability.

(2) $$\propto_{{CO}_{2}/{CH}_{4}}=\frac{P_{{CO}_{2}}}{P_{{CH}_{4}}}\;\;$$

The solution-diffusion mechanism is widely accepted as the primary transport mechanism for gas permeation through a dense membrane [37,38]. In this mechanism, the gas solubility coefficient (cm^3^(STP)/(cm^3^ cmHg)) is calculated using Equation (3):

(3) $$S=\frac{P}{D}\;\;\;\;\;$$

where P represents permeability and D represents the gas diffusion coefficient (cm^2^/s). The gas diffusion coefficient was determined using the time-lag method described by Equation (4) [37,39]:

(4) $$D=\frac{l^{2}}{6\theta }\;\;\;\;$$

where D is the diffusion coefficient (cm^2^/s), l is the membrane thickness (cm), and θ is the diffusion time lag (s) determined by the linear portion of the curve intercepting the time axis.

## 3. Results

### 3.1. Characterization of the ILs

Figure S2 shows the FTIR spectra of the ILs. The IL [GLYMIM]Cl exhibited the expected bands confirming the desired product formation. The OH band between 3281 and 3239 cm^−1^ was visible, as well as bands related to the imidazolium ring near 1670 cm^−1^ (C=C), 1604 cm^−1^ (N-H), and 1371 cm^−1^ (aromatic C-N) [25,40]. The substitution of the Cl anion by NT_2_F resulted in the appearance of bands between 1200 cm^−1^ and 1400 cm^−1^, characteristic for this ion, as well as others near 1060 cm^−1^ corresponding to S=O bonds, 846 cm^−1^ corresponding to N-S bonds, 789 cm^−1^ corresponding to C-S bonds, and 751 cm^−1^ corresponding to C-F bonds. Substitution of Cl by BF_4_ and PF_6_ anions also caused changes in the spectra, resulting in the appearance of bands at 815 cm^−1^ for PF_6_ P-F bonds and at 1050 cm^−1^ for BF_4_ B-F bonds [41,42,43].

For illustrative purposes, the NMR spectra of the chloride-functionalized N-glyceryl-N-methylimidazole [GLYMIM]Cl (Figure S3) and [GLYMIM]NT_2_F (Figure S4) ILs, obtained after the exchange of the chloride anion (Cl^−^) for NT_2_F, are shown. The 1H NMR spectrum (DMSO-d6) of [GLYMIM]Cl (Figure S2) exhibited characteristic peaks: 3.4 ppm (2H, CH2), 3.7 ppm (1H, CH), 3.9 ppm (3H, CH3), 4.4 ppm (2H, CH2), 5.3 ppm (1H, OH), 5.6 ppm (1H, OH), 7.7 ppm (1H, CH), 7.8 ppm (1H, CH), and 9.2 ppm (1H, CH) [25,32,44]. The 13C spectrum (DMSO-d6) of [GLYMIM]NT_2_F (Figure S3) showed characteristic peaks: δ 36.1 (CH_3_); 52.5 (-N+CH_2_); 63.1 (CH_2_OH); 70.07 (CHOH); 123.5 (CH); 123.6 (CH); 137.5 (CH); and 117.4 (2C, NT_2_F) [25,32,44].

### 3.2. Characterization of Cationic Poly(Ionic Liquids)

**Figure 3:**
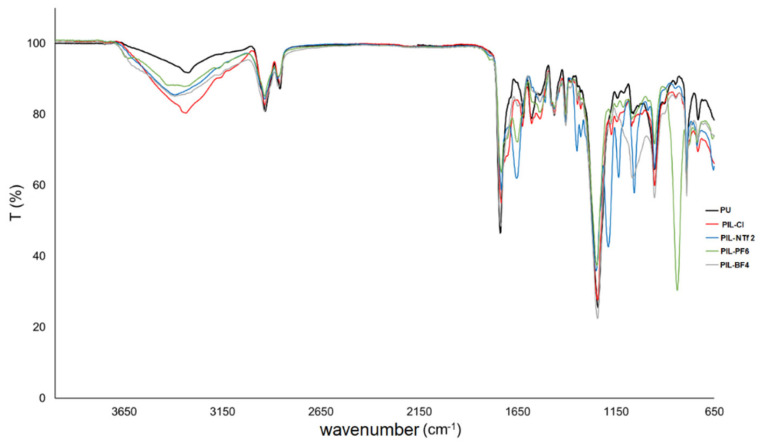
Infrared spectra of PU and cationic PILs.

**Figure 4:**
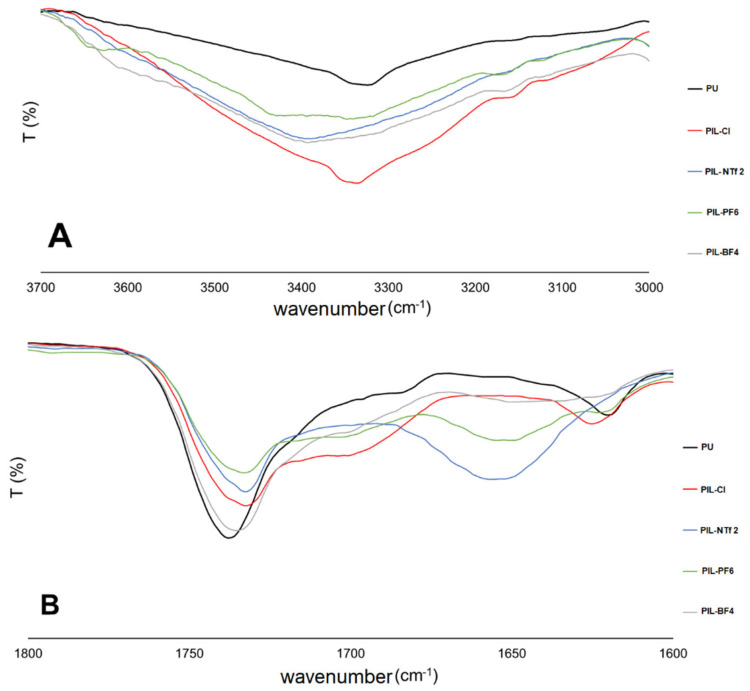
Infrared spectra of PU and cationic PILs. (**A**) 3000–3700 cm^−1^; (**B**) 1600–1800 cm^−1^.

The molecular weight distributions (PD, polydispersity) and the weighted molecular weights (Mw) of the PILs are presented in Table S1. The Mw values ranged from 43,264 gmol^−1^ to 70,655 gmol^−1^, while the polydispersity (PD) ranged from 1.2 to 1.4. In Figure 3, spectra obtained for the PILs are shown, and Figure 4 shows the enlargements of the spectra in the regions of 3000–3700 cm^−1^; 1600–1800 cm^−1^; and 650–1350 cm^−1^. FTIR spectra (Figure 3) showed characteristic bands found for polyurethanes: 2936–2871 cm^−1^ (CH_2_ and CH_3_ stretching), 1536 cm^−1^ (N–H bending groups), 1246 cm^−1^ (vibration of C-N and C-O groups of urethane), 1041 cm^−1^ (stretching of C–O–C groups of urethane), and 955 cm^−1^ (stretching of C–O–C groups of polycarbonate diol) [45]. The bands characteristic of H bonds present in polyurethanes were also evaluated: the region between 3200 and 3500 cm^−1^ corresponding to the stretching vibration of the N-H group of urethane (Figure 4A) and the region between 1700 and 1730 cm^−1^ characteristic of bonded and non-bonded carbonyl groups, respectively (Figure 4B). Shifts in characteristic bands (N-H and C=O) evidence possible H bonds. Figure 4A shows the characteristic bands of the stretching vibration of urethane N-H groups, where the region around 3400 cm^−1^ corresponds to non-bonded N-H groups and the band in the region of 3300 cm^−1^ characterizes the vibration of bonded N-H groups. All PILs showed an increase in intensity in the regions of bonded and non-bonded N-H groups with the insertion of IL into the polymeric chain. Also, concerning the structure of polyurethane, Figure 4B contains an enlargement of this area between 1600 and 1800 cm^−1^. In this range, it is possible to observe bands related to non-bonded carbonyl groups near 1730 cm^−1^ and bonded carbonyl groups near 1700 cm^−1^ [27]. In Figure 4B, changes in the spectrum were also noticed only when IL insertion is performed, such as the displacement of the band ~1730 cm^−1^ to values close to ~1710 cm^−1^. The appearance of characteristic bands of bonded carbonyl groups in the regions of 1700 cm^−1^ for samples with Cl, PF_6_ and BF_4_, and 1650 cm^−1^ for samples with NTf_2_, PF_6_, and BF_4_ counterions was observed, indicating the formation of H bonds [26,27]. The absence of the characteristic band for the stretching vibration of the N=C=O group in the region of 2270 cm^−1^ confirms the absence of free NCO groups in the polymeric material, indicating the formation of PU [27,29,46]. Bands related to the counter anions present in the samples can also be visualized (Figure 3). The PIL-BF4 sample presents a broad band near 1060 cm^−1^, indicating the presence of the B-F bond [41,42,43]. The PIL-PF_6_ presents a band near 835 cm^−1^ related to P-F bonds. For PIL-NTf_2_, a band near 1346 cm^−1^ related to SO_2_ bond vibrations was observed, as well as bands near 1136 cm^−1^ and 1189 cm^−1^ related to C-F bond vibrations [41,42,43].

#### 3.2.1. DSC

**Figure 5:**
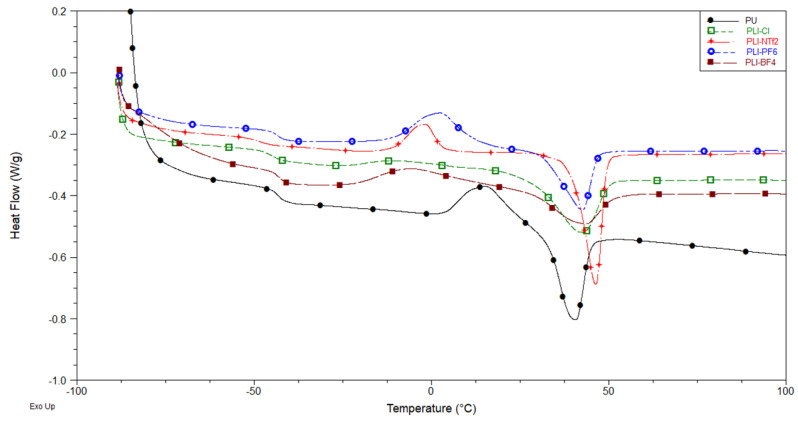
DSC curves of the PILs.

The DSC curves obtained for the polymeric samples are presented in Figure 5. The glass transition temperatures (Tg) values of −44.7, −47.9, −43.2, and −42.7 °C were found for the samples PIL-Cl, PIL-NTf_2_, PIL-PF_6_, and PIL-BF_4_, respectively. By comparing the values obtained for the PILs with the result obtained for pure PU (−42 °C), it is possible to perceive a decreased tendency in the Tg values for all PILs in relation to the non-ionic PU, which could mean that the addition of ILs in the polymeric chain increases the separation of the polymer microphases. This separation of microphases facilitates the mobility of the chains [26,27]. From the analysis of the thermograms, it is possible to identify the existence of an endothermic peak which can be attributed to the melting of a crystalline microphase (Tmf). This endothermic peak is characteristic for PU-based polymers that use polyol PCD with a molecular weight of 2000 g/mol in the polymer chain [26,27]. The results found for the PU, PIL-Cl, PIL-NTf_2_, PIL-PF_6_, and PIL-BF_4_ samples were, respectively, 40.1 °C, 42.2 °C, 46.4 °C, 42.8 °C, and 43.5 °C. Finally, the crystallization temperature (Tc) was obtained for the PIL-Cl sample (−12.7 °C).

#### 3.2.2. TGA

Table 1 and Figure S5 present the results of the degradation temperature obtained by TGA. PIL samples showed their first degradation temperature between 279 °C and 325 °C. This initial decomposition temperature corresponds to the rigid segments, where the degradation of the polyurethane bonds occurs. The second stage occurs around 440–469 °C, which refers to the breakdown of the flexible segments, leading to the degradation of the polyol along with the imidazole molecules. The incorporation of ILs led to an increase in the decomposition temperature, which may be related to the hydrogen bonds that may occur between PU and IL. Thermal decomposition of ILs typically occurs between 200 and 350 °C, suggesting that the decomposition of ILs may be occurring in the first stage of PILs degradation [47,48,49].

#### 3.2.3. DMA

**Figure 6:**
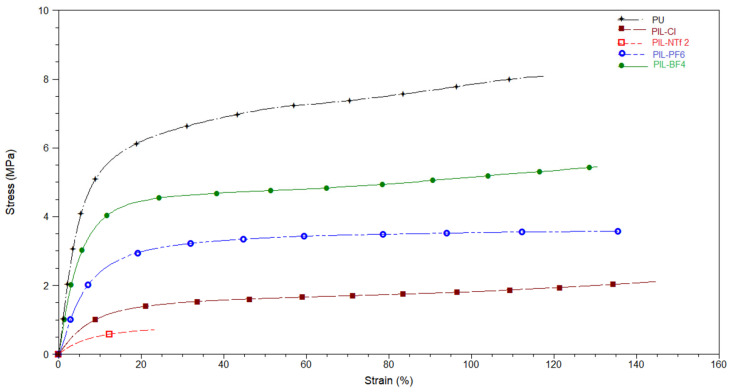
Stress–strain curves of PU and cationic PILs.

Figure 6 shows the stress–strain curves referring to the PILs samples. In terms of Young’s modulus, the increasing order is PIL-NTf _2_ < PIL-Cl < PIL-PF_6_ < PIL-BF_4_ < PU. The Young’s modulus values obtained for the PILs samples were 7 MPa (PIL-NTf_2_), 16.4 MPa (PIL-Cl), 29 MPa (PIL-PF_6_), 47 MPa (PIL-BF_4_), and 53 MPa (PU). The insertion of an IL into the polymeric chain can act as a plasticizer, interfering with the flexibility and mobility of the chains, affecting the Young’s modulus and resulting in its decrease [27,47]. Another possibility for this material behavior is the increased separation of the polymer microphases, which can also lead to a decrease in the Young’s modulus and elongation of the curve [50,51]. Except for PIL-NTf_2_, it can be observed that, besides the decrease in Young’s modulus, which indicates greater elasticity of the material, the curve becomes more elongated when comparing the PIL samples with the non-ionic PU sample. 

### 3.3. CO_2_ Sorption Capacity 

**Figure 7:**
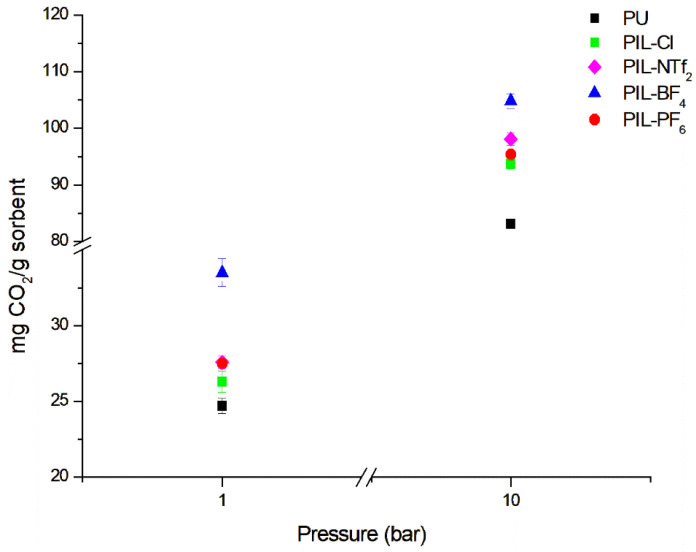
Sorption results for PU and cationic PILs at 1 and 10 bar with a temperature of 303.15 K.

The CO_2_ capture results can be observed in Figure 7. From the values found, it can be noticed that increasing pressure also increases the amount of captured CO_2_. This behavior is typical of samples that undergo CO_2_ sorption through physical interaction, as is the case with the PILs obtained in this work. The results obtained for the non-ionic PU sample were 24.7 mg CO_2_/g (1 bar) and 83.1 mg CO_2_/g (10 bar). These CO_2_ capture results before the addition of the ionic liquid can be attributed to the interactions between CO_2_ and the polar groups composed of nitrogen and oxygen present in the structure of the PU polymer chain. Additionally, Figure 7 shows that the insertion of the IL leads to a slight increase in CO_2_ capture values for all PIL samples. The results obtained for 1 bar were 27.5 mg CO_2_/g for PIL-PF_6_, 26.3 mg CO_2_/g for PIL-Cl, 27.6 mg CO_2_/g for PIL-NTf_2_, and 33.5 mg CO_2_/g for PIL-BF_4_. For the same samples at 10 bar, the results were 93.7 mg CO_2_/g for PLI-PC-Cl, 95.4 mg CO_2_/g for PLI-PC-PF6, 98.1 mg CO_2_/g for PIL-NTf_2_, and 104.8 mg CO_2_/g for PIL-BF_4._ The sorption capacity increases in the following order: [Cl]^−^ < [PF_6_]^−^ < [NTf_2_]^−^ < [BF_4_]^−^ for 1 and 10 bar. As expected, fluorinated anions showed a higher affinity for CO_2_. However, in this case, the size of the anion also appears to influence the CO_2_ sorption capacity. The structures of the NTF_2_^−^ and PF_6_^−^ anions are larger when compared to the BF_4_^−^ [41,52]. This may block some important CO_2_ interaction sites in the PIL cation, reducing the sorption capacity [53]. In the tests conducted, the CO_2_ sorption capacity of PIL-BF_4_ was superior compared to PU-based PILs reported in the literature using the BF_4_ anion. For example, Morozova 2017 [29] and Morozova 2020 [28] obtained 24.7 mgCO_2_/g for the PIL-8.1.BF_4_ and 24.8 mgCO_2_/g for PUR2.BF_4_ at 1 bar and 273 K.

### 3.4. Permeability and CO_2_ Selectivity 

Table 2 presents the results of the CO_2_ permeability and ideal (CO_2_/CH_4_) selectivity obtained for dense membranes of cationic PILs and pristine PU, compared to the results of cellulose acetate and some PILs found in the literature. The incorporation of ILs into the polymer chains produced membranes with superior performance when compared to neat PU, as seen in Table 2. It is observed that PIL-CL and PIL-BF_4_ samples tested exhibited higher CO_2_/CH_4_ selectivity than cellulose acetate [54,55]. Furthermore, it can be highlighted that the PIL-BF_4_ sample showed the best permeability and selectivity results among all samples presented in Table 2. The PIL-NTf_2_ sample presents poor mechanical properties, breaking during the test. Better CO_2_ permeability is expected for PILs, as the diffusion and solubility of CO_2_ molecules are generally high in ionic liquids [56]. The influence of PIL anions on CO_2_ affinity is well documented in the literature [53] and corroborated in our experiments. Permeability and selectivity increased when the Cl^−^ anion was replaced by BF_4_^−^, indicating that the presence of fluorine in PILs can enhance CO_2_ affinity. However, interestingly, the PF_6_^−^ anion showed lower permeability and selectivity compared to the other anions. This behavior may be related to the size of the anion. The sizes of the anions used in this work follow the general trend Cl^−^ < BF_4_^−^ < PF_6_^−^ <NTF_2_^−^ [41,52]. Therefore, although BF_4_^−^ and PF_6_^−^ have similar structures, PF_6_ has a larger size. Bulky anion structures can reduce the free volume of the PIL, hindering CO_2_ penetration towards the cation, which is primarily responsible for sorption [53]. According to Vollas et al. [41], the presence of smaller anions such as BF4^−^ facilitates polymer packing through chain interactions, leading to denser structures and consequently lower methane permeabilities. Therefore, there appears to be an optimal size for fluorinated anions (BF_4_^−^) to achieve high permeability and selectivity.

To obtain more information about the gas transport mechanism through the membranes, the diffusion and solubility coefficients for CO_2_ were determined. These coefficients can provide a better understanding of the variations in permeability obtained for the different PILs, as presented in Table 2. Furthermore, these coefficients can offer a better understanding of the permeability variations of PIL membranes with different counter anions, as presented in Table 3. The values of the diffusion and solubility coefficients showed that the obtained PIL membranes exhibited higher diffusivity and solubility to CO_2_ than to CH_4_, which contributed to the achieved CO_2_ permeabilities. Additionally, this behavior may be related to the high affinity of ILs with the polar groups of CO_2_. It can be highlighted that the PIL-BF_4_ sample showed higher diffusion and solubility coefficients, corroborating with the sorption capacity results. It is known that CO_2_ solubility is influenced by various factors, such as the size of the anions, which can lead to an increase in the free volume of PIL, thus enhancing gas solubility since there is more space available for sorption [63]. 

### 3.5. Comparison with Robeson Upper Bound

**Figure 8:**
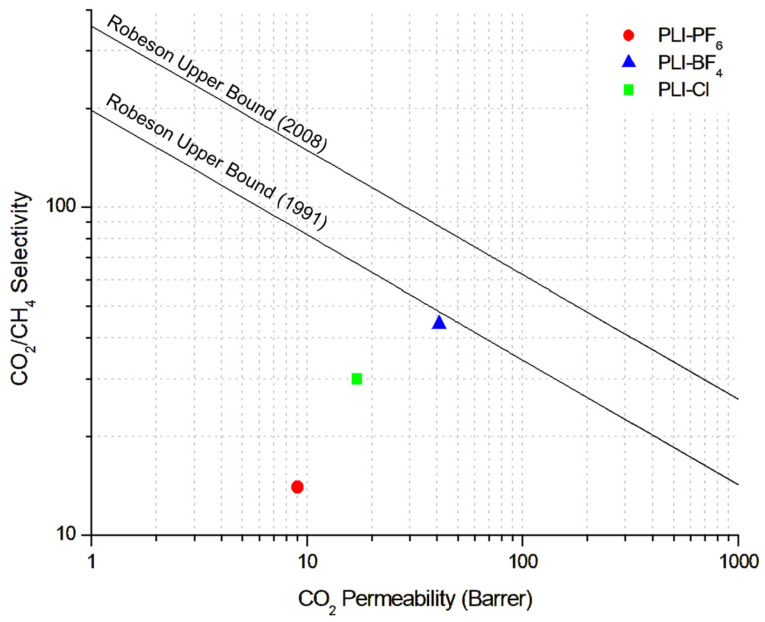
Performance of cationic PIL membranes at the Robeson upper bound for CO_2_/CH_4_ separation.

The separation performance of dense cationic PIL membranes in this study was compared with Robeson’s curves for CO_2/_CH_4_ [64,65], as shown in Figure 8. It can be observed that the membranes achieved performance below the Robeson limit; however, the PIL-BF_4_ sample is better positioned when compared to samples with Cl^−^ and PF_6_^−^ anions, being very close to the upper limit of 1991. A likely explanation lies in the relatively low CO_2_ permeability of the PILs with Cl^−^ and PF_6_^−^ anions, although they show superior results compared to commercial polymer cellulose acetate. The highlight of the PIL-BF_4_ sample is the ideal selectivity for CO_2_/CH_4_, which is higher than in other studies reported in the literature [66].

**Figure 9:**
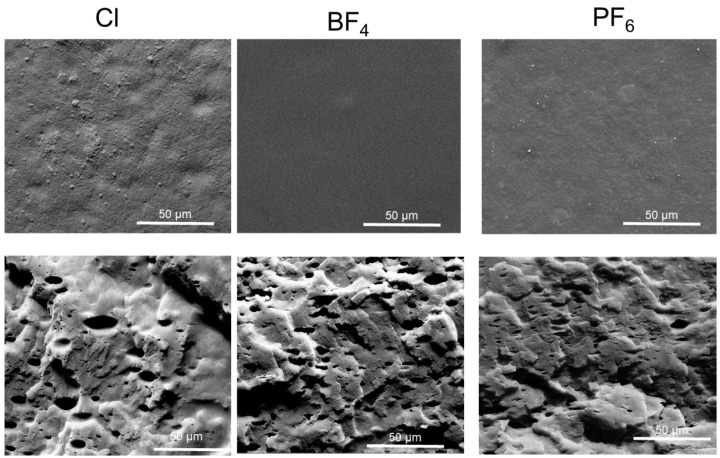
Surface and cross-sectional micrographs (cryogenic fracture) of PIL membranes, magnification, 3000×.

In this context, the obtained permeability and ideal selectivity results demonstrate that the synthesized PIL membranes are extremely promising for CO_2_/CH_4_ separation, especially because there is an intention to explore structural modification of the polymer chain to enhance the permeability and selectivity of these membranes [41]. It can be observed that the samples of cationic PILs prepared with different counter anions exhibited good permeability and high selectivity for CO_2_. From the SEM images shown in Figure 9, it can be seen that the PIL membranes produced for the permeability tests exhibited a surface without pores and defects.

## 4. Conclusions

In this study, we successfully developed PIL membranes that exhibited adequate thermal and mechanical properties, high CO_2_ permeability, and ideal CO_2/_CH_4_ selectivity. The IL GLYMIM [Cl] was synthesized and thoroughly characterized, and different counter anions’ chemical structures (NTf_2_, PF_6_, and BF_4_) were evaluated to identify the optimal combination for physicochemical properties, sorption, and permeability. FTIR analysis confirmed the presence of the characteristic bands for both ILs and PILs. The incorporation of IL into the polymer chain resulted in a decrease in the Tg values, indicating improved membrane flexibility. Among the samples tested, PIL-BF_4_ demonstrated the best performance in terms of sorption, permeability, and ideal CO_2_/CH_4_ selectivity. This suggests that appropriately sized fluorinated anions significantly enhance CO_2_ permeability and ideal CO_2/_CH_4_ selectivity, outperforming the commercial cellulose acetate membrane.

## Figures and Tables

**Table 1:** Results of degradation temperatures obtained by TGA.

Sample	T_onset1_ (°C)	T_onset2_ (°C)
PU	276	428
PIL-Cl	279	469
PIL-NTf_2_	318	462
PIL-PF_6_	300	440
PIL-BF_4_	325	445

**Table 2:** Permeability and ideal selectivity results for cationic PILs (25 °C and 4 bar), compared with results for cellulose acetate and PILs from the literature.

Membrane	Perm. CO_2_ (Barrer)	Perm. CH_4_ (Barrer)	Sel. Ideal CO_2_/CH_4_
PU	2.0 ± 0.1	0.78 ± 0.02	2.5
PIL-Cl	17 ± 2.2	0.6 ± 0.1	30
PIL-NTf_2_	-	-	-
PIL-PF_6_	9 ± 1.7	0.6 ± 0.2	14
PIL-BF_4_	41 ± 3.2	0.9 ± 0.3	44
1—CTA (25 °C, 5 bar)	18	1.98	9
2—CA (25 °C, 3 bar)	15	1.45	10
3—CA (25 °C, 1 bar)	4	0.2	17
4—CA (35° C, 3 bar)	4.3	0.21	21
5—Styrene-Based Poly(RTILS) (2 atm; RT)	9.2 ± 0.5	0.24 ± 0.01	39
5—Acrylate-Based Poly(RTILS) (2 atm; RT)	7.0 ± 0.4	0.19 ± 0.02	37
6—PIL-TFSI (1 atm; RT)	4.1 ± 0.1	-	41
7—OEG_1_ (2 atm; 295 K)	16 ± 1	0.48 ± 0.01	33
7—OEG_2_ (2 atm; 295 K)	22 ± 1	0.74 ± 0.02	29
8—PIL-i-propyl (3 atm; 20 °C)	10.4 ± 0.2	0.35 ± 0.01	30
9—Poly[DPyDBzPBI-BuI][Tf2N] (20 atm; 35 °C)	36.2	1.25	29

1—Raza, A. et al., 2021 [54]; 2—Mubashir, M. et al., 2018 [55]; 3—Saijan, P. et al., 2020 [57]; 4—Akbarzadeh, E. et al., 2021 [58]; 5—Bara, J. et al., 2007 [59]; 6—Vollas, A. et al., 2018 [41]; 7—Bara, J. et al., 2008 [60]; 8—Horne, W. et al., 2015 [61]; 9—Shaligram, S. et al., 2016 [62].

**Table 3:** Diffusion and solubility coefficients for PIL membranes at 4 bar and 25 °C.

Membrane	D (10^−8^ cm²/s)		S (10^−2^ cm³(STP)/(cm³ cmHg))		D_CO2/CH4_	S_CO2/CH4_
	CO_2_	CH_4_	CO_2_	CH_4_		
PIL-Cl	21.60	2.32	0.78	0.26	9.32	3.04
PIL-NTf_2_	-	-	-	-	-	-
PIL-PF_6_	22.04	1.61	0.41	0.37	13.68	1.10
PIL-BF_4_	32.67	1.79	1.26	0.50	18.22	2.52
CA (35 °C, 3 bar) [57]	2.05	0.55	2.11	0.38	3.72	5.55

## Data Availability

The original contributions presented in the study are included in the article, further inquiries can be directed to the corresponding author.

## Supplementary Materials

The following supporting information can be downloaded at: https://www.mdpi.com/article/10.3390/membranes14070151/s1, Figure S1: Illustration of permeability system set up; Figure S2: Synthesized ionic liquids spectra; Figure S3: IL ([GLYMIM]) Cl RMN spectra, 1H (DMSO-d6); Figure S4: IL [GLYMIM]NT2F 13C RMN spectra; Figure S5: PILs TGA curves; Table S1: PILs GPC results.

## Nomenclature

PILs	poly(ionic liquids) or polymerized ionic liquids
ILs	ionic liquids
GLYMIM	glyceryl-N-methylimidazolium
Cl^−^	chloride anion
NTf_2_^−^	bis(trifluoromethanesulfonyl)imide anion
PF_6_^−^	hexafluorophosphate
BF_4_^−^	tetrafluoroborate
PU	polyurethane
PTMG	polytetramethylene glycol
PCD	polycarbonate diol
PCL	polycaprolactone
HDI	hexamethylene diisocyanate
DBTDL	dibutyltin dilaurate
MEK	methyl ethylketone
PD	polydispersity
Mw	molecular weights
Tg	glass transition temperatures
