# Supplementary material for: Cationic Imidazolium-Urethane-Based Poly(Ionic Liquids) Membranes for Enhanced CO2/CH4 Separation: Synthesis, Characterization, and Performance Evaluation
Source: Membranes (Basel). 2024 Jul 9;14(7):151. doi: 10.3390/membranes14070151 (PMC11279342; doi:10.3390/membranes14070151)
Supplement: Supplementary file 1 [file membranes-14-00151-s001.zip › membranes-3062448-supplementary.pdf]

Cationic Imidazolium-Urethane Based Poly(ionic liquids) Membranes for Enhanced CO_2/CH_4 Separation: Synthesis, Characterization, and Performance Evaluation

Guilherme Dias[1,2], Laura Rocca[1], Henrique Z. Ferrari[1,2], Franciele L. Bernard[1], Fernando G. Brandão[3], Leonardo Pereira[3], Sandra Einloft[1]

[1]School of Technology, Pontifical Catholic University of Rio Grande do Sul (PUCRS), Avenue Ipiranga, 6681, Partenon, CEP: 90619-900, Porto Alegre, Brazil

[2]Post-Graduation Program in Materials Engineering and Technology, Pontifical Catholic University of Rio Grande do Sul (PUCRS), Porto Alegre, Brazil

[3]Petrobras/CENPES, Ilha do Fundão Qd. 07, Rio de Janeiro, RJ, Brazil

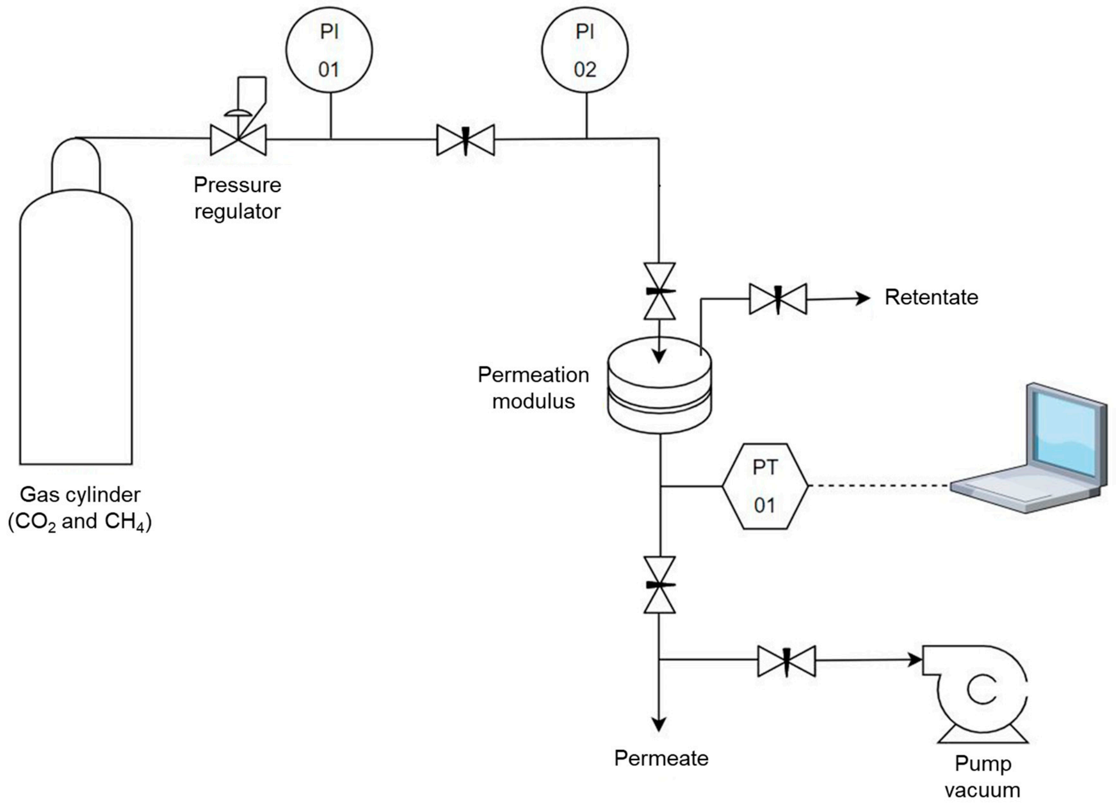

Figure S1. Illustration of permeability system set up.

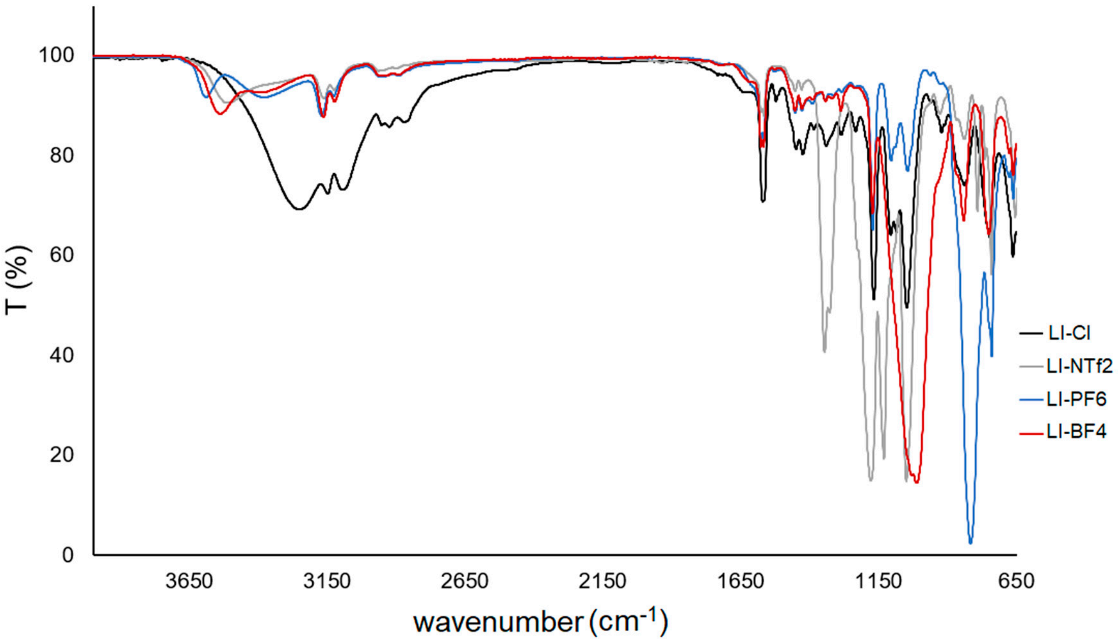

Figure S2. Synthesized ionic liquids spectra.

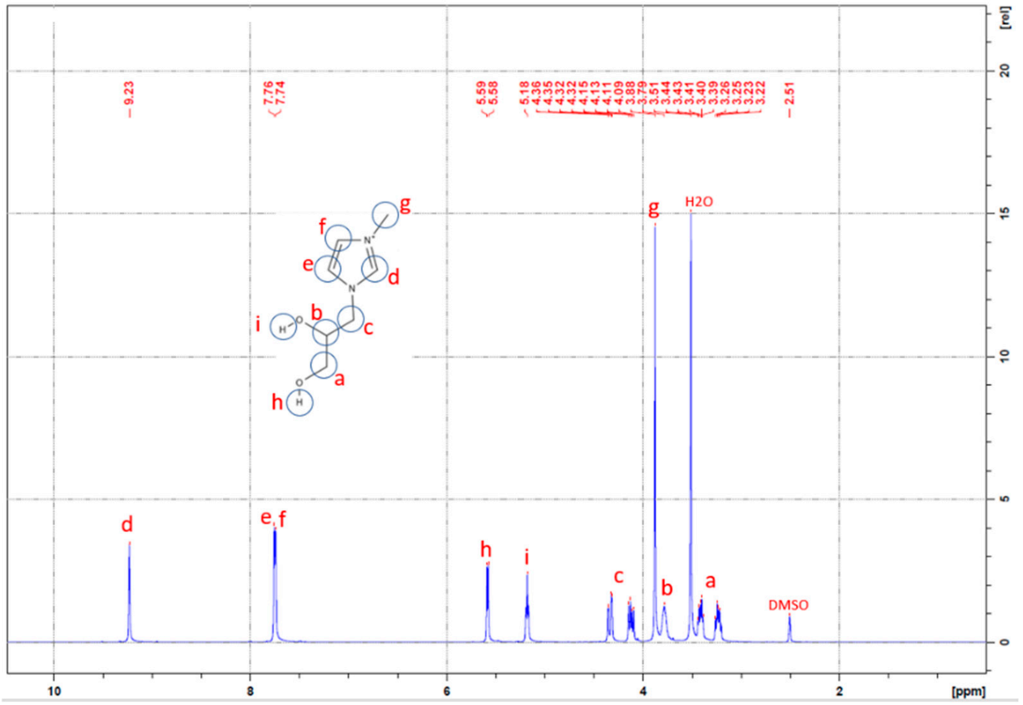

Figure S3. IL ([GLYMIM]) Cl RMN spectra, 1H (DMSO-d6).

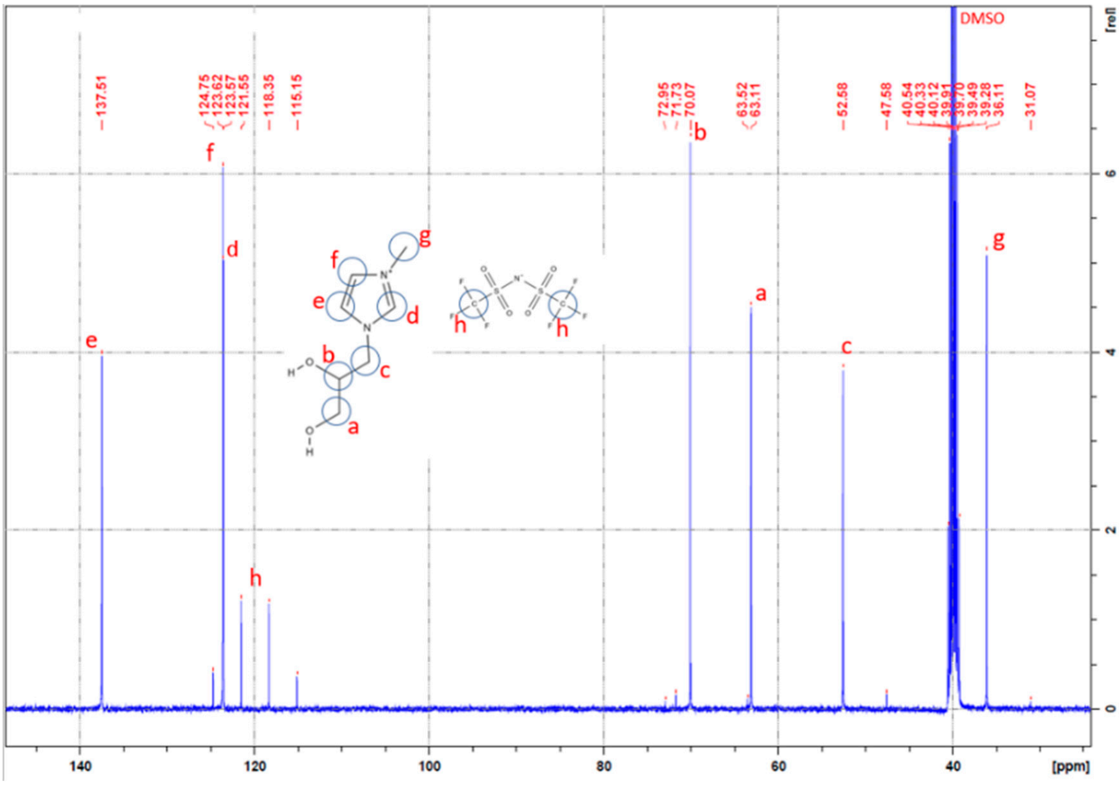

Figure S4. IL [GLYMIM]NT2F 13C RMN spectra.

Table S1. PILs GPC results.

Samples	Mw (gmol^{-1})	PD
PIL-Cl	48311	1,36
PIL-NTf$_2$	45485	1,22
PIL-BF$_4$	43264	1,42
PIL-PF$_6$	70655	1,24

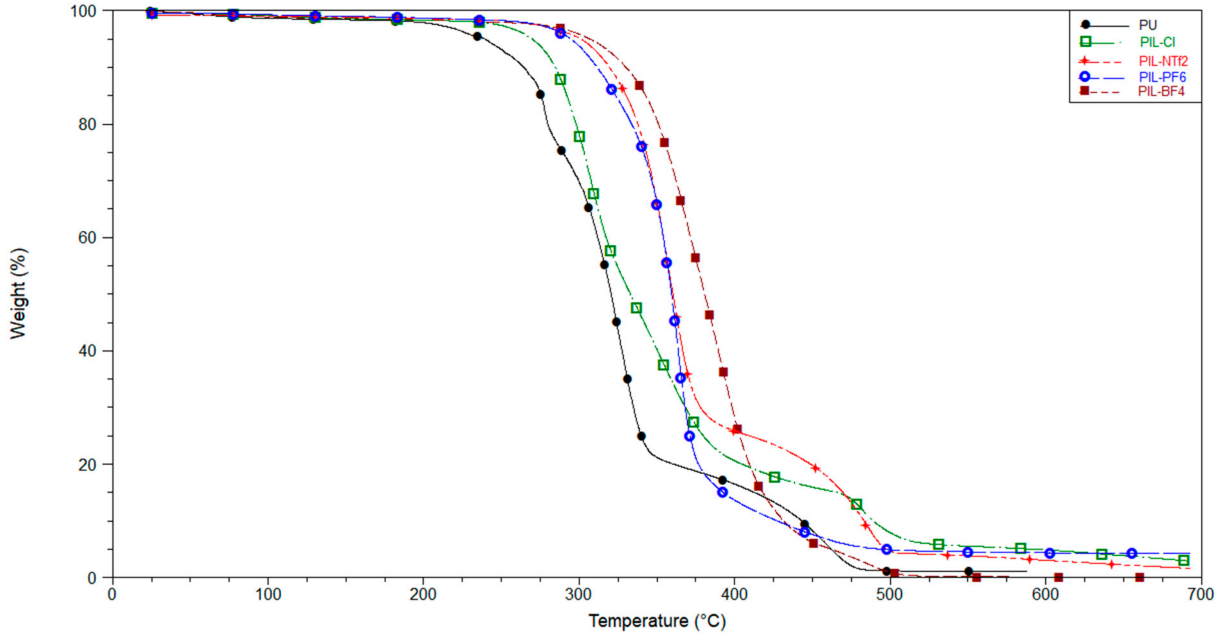

Figure S5. PILs TGA curves.
